# Pediatricians’ perceptions of gender issues that influence the choice of medical specialty

**DOI:** 10.47626/1679-4435-2022-1002

**Published:** 2023-11-24

**Authors:** Mônica Ramos Daltro, Pedro Guerra Júnior, Luiza Rodrigues Santos

**Affiliations:** 1 Escola Bahiana de Medicina e Saúde Pública, Salvador, BA, Brazil

**Keywords:** pediatrics, gender and health, gender inequality, pediatria, gênero e saúde, iniquidade de gênero

## Abstract

**Introduction:**

The medical field has witnessed a growing expansion of the female workforce. Despite a greater appreciation and trust in women in pediatric spaces, gender violence remains a significant concern on the health agenda.

**Objectives:**

This study aimed to investigate how pediatricians perceive gender experiences in daily work, discussing its effects on professional practice.

**Methods:**

The study adopted a descriptive, exploratory, and qualitative approach and was conducted in a university hospital with 14 pediatricians from the urgency and emergency service. Virtual semi-structured interviews were conducted to explore sociodemographic data, training, and professional background. The narratives obtained were subjected to Content Analysis, resulting in the construction of five categories: choice by specialty; maternity; place of reception; violence; and the male doctor.

**Results:**

It was observed that pediatricians build their professional routine based on conservative values of patriarchal and sexist anchorage.

**Conclusions:**

The set of narratives emphasize the importance of confronting gender inequality within the scope of medical training.

## Abstract (pt)

**Introdução:**

A medicina vem apresentando uma crescente expansão da mão de obra feminina. Apesar de maior valorização e confiança em mulheres nos espaços pediátricos, as violências de gênero fazem parte da agenda de saúde.

**Objetivos:**

Investigar como os pediatras percebem as vivências de gênero no cotidiano do trabalho, discutindo os seus efeitos na prática profissional.

**Métodos:**

Tratou-se de um estudo descritivo, exploratório e qualitativo, realizado em um hospital universitário com 14 pediatras do serviço de urgência e emergência. As entrevistas semiestruturadas foram realizadas virtualmente, investigando dados sociodemográficos e percursos formativo e profissional. As narrativas foram analisadas pelo método de análise de conteúdo. Foram formadas cinco categorias: escolha pela especialidade; maternidade; lugar de acolhimento; violências; e o médico homem.

**Resultados:**

Observou-se que pediatras constroem seu cotidiano profissional a partir de valores conservadores de ancoramento patriarcal e machista.

**Conclusões:**

O conjunto das narrativas coloca a importância do enfrentamento da desigualdade de gênero no âmbito da formação médica.

## INTRODUCTION

Throughout its history, medicine has been practiced predominantly by men. However, although men still comprise most of the physicians in Brazil, the medical profession has witnessed a notable increase in the presence of women, indicating a clear trend toward the feminization of medicine in the country.^1,2^

According to the Medical Demography in Brazil, the percentage of male physicians in 2020 was 53.4%, compared to 57.5% in 2015 and 69.2% in 1990. An analysis of the data by age group reveals that women predominate among younger doctors, corresponding to 58.5% of doctors up to 29 years old and 55.3% of doctors aged 30 to 34. However, between ages 35 and 39, there is a balance between the sexes, with women accounting for 49.7% of doctors in this age range.^2^

Regarding specialties, pediatrics stands as the second medical area with the highest proportion of female specialists (74.4%).^2^ This trend may reflect some aspects related to identity, personality, and behaviors structured by historical processes in women.^1^ Research has revealed that valuing psychosocial factors, patient-centered care, and an emphasis on cultivating stronger doctor-patient relationships, allowing for the development of long-term relationships, are more prevalent among women.^3-5^

Indeed, the socialization process differs between men and women, which may result in different tendencies and approaches to care and communication within the healthcare practice context. The literature supports these observations, demonstrating how these perspectives are manifested in the clear preference for women practitioners when parents and children choose a pediatrician.^6^

The figure of the woman professional may be linked to higher levels of parental trust. This is attributed to the perceived greater potential of women to interact with children and families with empathic attitudes, which are often more prevalent in women.^6-8^ As a result, this reality fosters increased recognition and trust in women within pediatric settings.^6^

Among the myths built by patriarchy about women, motherhood as a fictional category was the most widespread and naturalized, defining women’s identity as circumscribed to the idea of care, sensitivity, and selflessness. This notion of motherhood as an institution sustains reductionist and harmful effects on women’s professional lives, engendering hierarchical structures and reinforcing a gender system that promotes violence and disqualification.^9^

Gender-based violence is part of the health agenda, so it is crucial to analyze its impact on the daily lives of pediatric health professionals. This form of violence stems from patriarchal culture and is often institutionalized and reinforced by its agents. Consequently, it is necessary to investigate how pediatricians perceive gender-related experiences in their everyday work and to discuss the implications of these experiences on their professional practice.

## METHODS

This was a descriptive, exploratory, qualitative study conducted at a university hospital in northeastern Brazil. Participants included all 14 pediatricians working in the pediatric emergency care service, who responded to semi-structured virtual interviews conducted between February and April 2021. The interviews focused on investigating sociodemographic data (age, sex, self-declared color, marital status, place of birth, and number and age of children), as well as exploring training and professional backgrounds. The narratives obtained from the interviews were analyzed using the content analysis method.^10^ The inclusion criteria for participants were physicians with a specialist title in pediatrics, admitted by public tender at the hospital and working in the pediatric emergency care service. Physicians who were unable to participate due to health reasons or lack of availability were excluded from the study.

The research was approved by the Research Ethics Committee of the Universidade Federal de Sergipe (CEP/UFS), under number 4.530.759, and all participants provided signed Informed Consent Forms. The names of characters from the literary works of author Jorge Amado were used in the research, which depicts empowered women willing to make changes in their communities.

## RESULTS

The analysis of the narratives using content analysis identified five thematic categories, which are presented in Chart 1 and discussed sequentially in an interrelated manner.

**Chart 1:** Thematic categories of pediatricians’ perceptions of gender issues

Pediatricians’ perceptions				
Choice of specialty	Maternity	Place of welcoming	Violence	The male physician

## DISCUSSION

The study population comprised 14 pediatricians, including 3 men and 11 women, with ages ranging from 29 to 53 years. Most pediatricians reported being black (brown and black), married, and without children.

Studies have shown that gender influences the choice of medical specialty, with men preferring general surgery and orthopedics, while women prefer pediatrics, obstetrics, and gynecology.^11-13^ However, in the present study, participants did not mention the influence of gender when asked about their motivation for choosing pediatrics.

Pediatrics may attract women because of the historical-cultural association of caring with motherhood, which is mentioned as a contributing factor in choosing pediatrics:

I’ve always wanted to be a mother, I’ve always loved children, and I think that made my choice much easier. (Lívia)

The image of women as caregivers is projected in their role in childcare. The socio-historical and cultural construction of the mother-woman portrays her as the bearer of informal knowledge associated with the idea of mandatory motherhood. This perception reduces women to a reproductive role and the responsibility for child-rearing, a premise that serves to keep them in the domestic sphere, even when they hold positions in the public world.^9^

It is important to analyze the place of paternity in this predominantly female professional setting, where the experience of fatherhood is excluded from the idea of care. In addition, the practical experience of the professionals highlighted in this study indicated that it is mainly women who choose the doctor and accompany their children to the pediatrician and that they often express a preference for their children to be seen by women pediatricians rather than male ones. Regarding the influence of gender on the choice of pediatrician, one participant provided the following explanation:

It does influence in some ways. It influences because most caregivers who bring children to the hospital are women, and we can empathize, as I said, attempting to put ourselves in their shoes, or at least strive to understand the maternal perspective. Our feminine sensitivity aids us in understanding the daily routines and experiences of women within their families. (Dora)

This cultural pattern that distances men from caregiving functions is transmitted across different societies and generations. It is supported by informal and formal educational processes, perpetuating sexual hierarchy and gender inequality, particularly regarding childcare. This research points out that such discursive logic is present in everyday life, making it undoubtedly a challenge to be faced by medical training.^14^

The association of the maternal figure with the professional pediatrician is also perceived by another interviewee, revealing that there is a kind of mirroring between the experience of motherhood and professional practice. This observation reaffirms the association between motherhood, femininity, and the caregiving aspect within the pediatric field:

In pediatrics you have to be patient with the mother, don’t you? [...] If I was already patient with mothers, after I became a mother, my patience increased even more. (Gabriela)

The narratives collected also mention empathy as an essential characteristic in pediatrics, as a strategy to improve communication with both the child and their family. Research indicates that being a married woman is an important predictor of empathy for physicians and medical students.^7,8^ This idea shows how entrenched patriarchal culture is in our society, which often leads to the automatic exclusion of professionals who do not fit this pattern. The result is a medical practice that is based on exclusionary elements that downplay the technical role of women’s work.

Reports on the significance of empathy in the professional context were made only by women, mostly married or in civil unions:

[...] I think my communication is pretty good, even because I put myself in the person’s shoes, I try to, and I understand that the child and the family are in a vulnerable situation. (Don’Ana)

In the perception of the interviewees, being a woman enhances their understanding of the patient’s reality, as they are attentive and sensitive towards situations of vulnerability, particularly concerning the mother and child. This fosters a sense of identification and connection. However, in a hetero-patriarchal society, these skills and qualities conventionally associated with femininity become preferred criteria in selecting a professional, influencing the doctor-patient relationship. This pattern reveals how inequality operates regarding the preference for a particular gender in various functions. It also illustrates the technical, social, and political segregation of work that contributes incisively to the devaluation of certain professions. On the subject of exclusion within the profession, one participant explained:

I think women in pediatrics sometimes feel less excluded. That’s the impression I get. Because they come with the question “Do you have a child too?” So they want to know if you feel like them, you know? As this question is frequent, I don’t think that only the fact of being a mother, but for some mothers it counts, in the sense of “ok, she understands what I’m talking about.” (Eugênia)

According to the accounts provided, the significant rise in the number of women in pediatrics, in turn, is perceived by one of the participants as a positive aspect, as it makes female doctors feel less excluded. This suggests the existence of gender-based exclusion and discrimination in the daily experiences of these professionals, and pediatrics, despite its patriarchal contours, is a place of greater security. Furthermore, unlike what happens in other medical fields, pregnancy among pediatricians allows women to be recognized by their patients’ mothers, thereby making them feel valued and validated in their role as mothers.

The author Chimamanda Ngozi Adichie^15^ (p. 13-15) teaches that the problem of gender is the premise that it determines “how we should be, rather than recognizing how we are.” Therefore, women are increasingly stifled by the pressures imposed on them by our patriarchal society, which is resulting in an increased suffocation of their freedom to decide and be who they are.

In a sexist society, numerous women face barriers due to their gender, a perspective that is present in medicine, particularly when it comes to selecting specialities.^16^ The choice to pursue a profession is often based on values that reinforce the expectations of mandatory motherhood. This concept supports the societal expectation that women are inherently destined for motherhood, with their bodies naturally inclined towards reproduction. Consequently, this idea restricts women’s freedom of choice regarding motherhood.^9^

Pediatricians reported that their profession often carries stigmas due to a social construction related to gender inequality. This influences parents’ pursuit of care and ends up interfering in relationships with both the health care professionals and service users, leading to challenging situations. One participant explains:

Yes, I believe so. There is, whether we like it or not, a prejudice that is kind of rooted in society, that most pediatricians also end up being women. So, in our daily lives, we realize that, unfortunately… I don’t think it’s correct or adequate, I don’t think there is a difference in treatment, but often parents end up looking for female pediatricians. (Tieta)

Considering this narrative regarding mothers’ choices, it is essential to also take into account the significant number of solo mothers in Brazil who serve as breadwinners. These women often make choices based on their circumstances and the absence of paternal figures in their lives. According to the Brazilian Institute of Geography and Statistics (IBGE), in 2018, there were approximately 11.5 million women in Brazil who had to take on the responsibilities of single-handedly raising and caring for their children.^17^

Even in a field predominantly occupied by women, gender-based violence unfolds in professional relationships and care settings. There is an urgent need to adopt measures to curb workplace moral harassment, a form of violence that is revealed in recurring behaviors that offend the dignity of professionals, leading to harm or psychological distress due to abuse in the exercise of employment, position or function.

Despite the increase in the number of women in medicine in recent years and the advancement of the fight for gender equity in medical institutions, professionals still suffer gender discrimination.^18^ In relation to this situation, a pediatrician pointed out:

Yes, I encountered challenges in my role as a woman providing medical care for a child. There was one case where a father was present and tried to oppress me because I was a woman, you know? I have, at times, felt intimidated in this sense, of having a threat because he was a man and I was a woman… So, I think that, as a woman doctor, it must be much more difficult to be an orthopedist, for example, than to be a pediatrician. That’s exactly why I told you that most of the children’s caregivers are women, so it’s easier to maintain a dialog and not have the possibility of being subjected to violence as I reported to you. (Dora)

Strategies of gender-based violence in the workplace become evident in the treatment of women in an infantilized manner as if they were incapable of understanding more formal or even technical language. Moreover, they are subjected to disrespect, being disqualified, and facing questioning regarding their professional competence. For this reason, it is not uncommon for medical professionals to be discredited in other specialties due to their gender - which includes their bodies -, as illustrated by the following participant’s account:

There is still an understanding in society that the doctor is a man. And due to my [small] size and baby-face appearance… That’s why I introduce myself as a doctor, because when people look at me, they don’t think I’m old enough to be a doctor. And in the hospital, I’m even more hidden, wearing a cap, a mask, so everybody looks the same now, and the clothes are not identifiable. So today, there’s even more this need for me to introduce myself because there is no identification that I am the doctor. But even when I had identification, I still introduced myself because my appearance does not fit the stereotype of a doctor. At the University Hospital, we work with trainee doctors and, countless times, patients would address the trainees, especially the males, assuming that they were the doctors, while I was recognized as the student. (Tereza)

This statement reveals the need to deconstruct certain prejudices that associate the professional image with a figure traditionally represented by white men in medicine. This image excludes the potential for an inclusive professional environment that embraces diverse gender, age, and racial identities. Frequently, this social perception of physicians is reinforced by cultural norms that perpetuate gender, race, and class biases, resulting in exclusion, suffering and veiled or explicit forms of violence.

Gender is also an issue in the management of the clinic, as instructed in the manual “Guidance for adolescent consultation: clinical approach, ethical and legal guidelines as tools pediatric practitioners”, prepared by the Brazilian Society of Pediatrics (SBP),^19^ which states that, in the first consultation, it is essential to point out that the central person of that care is the child/adolescent, thus clarifying their rights in relation to confidentiality, privacy, and reliability. However, no handbook teaches about the limits of ethics in the process of physical touch, whose moral values can and should be taught in the context of academia, but require the establishment of a culture sensitive to ethical concerns, and this implies special attention to the place of gender differences in culture. Male participants expressed in their speeches that they feel the discomfort of some female patients when performing the physical examination:

Often, in the physical examinations, if we examine a girl, or have to examine the genitalia, there might be some apprehension from the child. But I make sure to create a comfortable environment for her. If she does not feel at ease, I say, “Your mother is going to take off your panties, I will examine to ensure everything is all right.” But if the child refuses, I don’t insist, and I say “Mother, it’s better for you to see a female doctor later, so that she can check this.” But it is very rare for that to happen, usually by the time they get there for the physical examination, they already feel more at ease, and boys or girls I don’t have any major problems examining, no. (Guma)

This narrative expresses the importance of respecting the limits of the body of the individual undergoing the physical examination, considering that there may be fear, shame, or discomfort when dealing with a male pediatrician. This report is extremely relevant for health care professionals; as such experiences may result in trauma for the patient, bringing psychological distress in the face of an experience that may lead to a memory of pain. Understanding the body as a field of subjective manifestations, marked by singular histories and experiences and acknowledging the prevalence of sexism in our culture aids in interpreting the body’s responses during an examination, particularly when conducted by a male.

The notion of men being perceived as potentially threatening and unwilling to listen is also ingrained in the daily life of male doctors:

Perhaps the aspect of being a man does have some influence, because, what I notice is, in pediatrics there are significantly more women than men, so many caretakers tend to prefer women. This doesn’t usually happen in my private practice, but in public service I’ve encountered situations where people say, “I don’t want to be seen by him because he is a man,” you know? And this saddens me greatly because they haven’t even given a chance to build trust; they simply block it. I find this to be quite unfortunate, but occasionally, it does occur. [...] I believe it’s more related to gender because of the predominance of women in pediatrics. They may end up preferring women, possibly due to the perception that women are more caring and approachable. Anyway, this is the only thing that I’ve noticed. (Antônio)

Gender bias poses a source of frustration for some male pediatricians, as they are often labeled according to a masculine standard that portrays the image of a provider father - authoritarian and solely responsible for the family’s financial support. This stereotype portrays them as incapable of displaying sensitivity, configuring the imaginary prototype of the one who exempts himself from other tasks of fatherhood, such as education, affection and care. Consequently, this stereotype perpetuates the disparity between the roles of men and women in patriarchal societies. As a result, the notion of fathers being active protagonists who provide care, affection, and assume responsibility in their children’s lives is automatically excluded from this conservative professional model.

An additional perspective that deserves attention involves the importance of discussing strategies focused expanding and promoting the inclusion and active participation of both fathers and mothers in childcare, with the aim of deconstructing the model centered on the exclusive involvement of the mother.^20^ As for the child, various factors may influence the preference for a female pediatrician. This difference is emphasized by the following participant:

It’s different, when children are seen by a male doctor, they react in a way, and when they are seen by a female doctor, they act in another way, but this is very personal. Each child is unique, and some may primarily receive care from women, so when a man arrives, they find it a bit strange, but the opposite also happens, though less frequently. (Pedro)

Pediatricians play a crucial role in supporting children’s development, respecting their limits, thus fostering self-confidence and self-esteem, and promoting positive relationships with other children, family, and community. However, the lack of identification with male pediatricians may be linked to various contexts, including instances of paternal emotional abandonment, experiences of abuse, or even the absence of references of male figures providing both physical and emotional care for the child.

## CONCLUSIONS

Brazilian society, despite recent discourses of moral and ethical regression, has advanced towards producing new and plural ways of living, and the search for gender equity in the field of health care is necessary. Gender is an issue constructed in the process of social interactions, and making sense of this issue is one of the challenges of everyday pediatrics.

The study found that pediatricians build their daily professional life on conservative values with patriarchal and sexist anchors, a perspective that is reflected in the patients’ view, according to the perception of the research participants. This study discusses how recognizing these elements can help in the management of different clinical situations.

The narratives as a whole emphasize the importance of tackling gender inequality education as a proactive action to promote a more equitable society and a clinical practice that can contemplate the ethical plurality of life.

## References

[1] Santos TS. (2010). Discriminações, estímulos e
obstáculos no campo profissional da medicina: um olhar de
gênero e gerações. Trab Educ Saude.

[2] Scheffer M. (2020). Demografia médica no Brasil 2020.

[3] Mendes AS. (2010). Os estudantes de medicina: expectativas na escolha da especialidade
[Dissertação de Mestrado].

[4] Corsi PR, Fernandes EL, Intelizano PM, Montagnini CCB, Baracat FI, Ribeiro MCSA. (2014). Fatores que influenciam o aluno na escolha da especialidade
médica. Rev Bras Educ Med.

[5] Osborn SR, Yu J, Williams B, Vasilyadis M, Blackmore CC. (2017). Changes in provider prescribing patterns after implementation of
an emergency department prescription opioid policy. J Emerg Med.

[6] Barbabela D, Mota JPT, Maia PGM, Bonanato K, Paiva SM, Pordeus IA. (2008). Preferência da criança pelo gênero do
odontopediatra. Arq Odontol.

[7] Katsari V, Tyritidou A, Domeyer P. (2020). Physicians’ self-assessed empathy and patients’ perceptions of
physicians’ empathy: Validation of the Greek Jefferson Scale of Patient
Perception of Physician Empathy. Biomed Res Int.

[8] Vogel D, Meyer M, Harendza S. (2018). Verbal and non-verbal communication skills including empathy
during history taking of undergraduate medical students. BMC Med Educ.

[9] Gonzaga PRB, Mayorga C. (2019). Violências e instituição maternidade: uma
reflexão feminista decolonial. Psicol Cienc Prof.

[10] Bardin L. (2016). Análise de conteúdo.

[11] Anand R, Sankaran PS. (2019). Factors influencing the career preferences of medical students
and interns: a cross-sectional, questionnaire-based survey from
India. J Educ Eval Health Prof.

[12] Kawamoto R, Ninomiya D, Kasai Y, Kusunoki T, Ohtsuka N, Kumagi T (2016). Gender difference in preference of specialty as a career choice
among Japanese medical students. BMC Med Educ.

[13] Alers M, Verdonk P, Bor H, Hamberg K, Lagro-Janssen A. (2014). Gendered career considerations consolidate from the start of
medical education. Int J Med Educ.

[14] Souza LP, Guedes DR. (2016). A desigual divisão sexual do trabalho: um olhar sobre a
última década. Estud Av.

[15] Adichie CN. (2014). Sejamos todos feministas.

[16] Nascimento MEFM, Niquen-Jimenez M, Campos LN, Ribeiro LLPA, Gois AFT. (2021). Promovendo equidade de gênero nas especialidades
cirúrgicas: experiência de programa de mentoria na
América Latina. Rev Bras Educ Med.

[17] Marques G [Internet] (2020). O abandono paterno e a culpabilização da
mulher. João Pessoa;.

[18] Ávila RC. (2014). Formação das mulheres nas escolas de
medicina. Rev Bras Educ Med.

[19] Sociedade Brasileira de Pediatria (2019). Consulta do adolescente: abordagem clínica,
orientações éticas e legais como instrumentos ao
pediatra.

[20] Lamy ZC. (2019). Participação do pai na unidade neonatal: um
processo em construção. Rev Paul Pediatr.

